# Cerebral Structural Abnormalities and Their Associations With Peripheral Cytokine Levels in a Group of Untreated Patients With Nasopharyngeal Carcinoma

**DOI:** 10.3389/fonc.2021.740033

**Published:** 2021-11-26

**Authors:** Yan Wang, Wenjing Zhang, Xin Wu, Xinmei Luo, Siyi Li, Guannan Zhu, Jie Liu, Qiyong Gong, Yu Jiang, Su Lui

**Affiliations:** ^1^ Huaxi MR Research Center (HMRRC), Department of Radiology, West China Hospital of Sichuan University, Chengdu, China; ^2^ Department of Radiology, the First Affiliated Hospital of Xi’an Jiaotong University, Xi’an, China; ^3^ Department of Medical Oncology, Cancer Center, West China Hospital of Sichuan University, Chengdu, China

**Keywords:** nasopharyngeal carcinoma, magnetic resonance imaging, brain structure, cytokine, depressive symptoms

## Abstract

The current study is to characterize the alterations of peripheral cytokines and anatomical brain changes, and their relationships in untreated nasopharyngeal carcinoma (NPC) patients with depressive symptoms. Twenty-nine newly diagnosed NPC patients without any treatment and 46 matched healthy comparisons were recruited, scanned with high-resolution T1 images and assessed psychologically using Hamilton Rating Scale for Depression (HAMD). Serum levels of interleukin-1 beta (IL-1β), IL-2, IL-6, IL-8, IL-10, interferon-gamma (IFN-γ) and transforming growth factor-beta (TGF-β) were measured by quantitative chemiluminescence assay. Inter-group comparisons of anatomical brain measures were performed, and regions with significant inter-group differences were correlated to HAMD scores and cytokines in NPC patients. A subgroup analysis especially within NPC patients with depression was conducted to precisely characterize the associations among serum cytokines, brain changes and depressive symptoms. Relative to healthy subjects, NPC patients showed significantly decreased cortical thickness in the left parahippocampal gyrus, increased surface area in the right superior parietal lobule and precentral gyrus, and increased gray matter volume in the right postcentral gyrus, bilateral caudate nucleus and right thalamus, as well as significantly elevated IL-1β, IL-2 and IL-10. The elevated IL-2 and IL-10 were negatively correlated with surface area in right superior parietal lobule, whilst IL-1β level was positively correlated to HAMD scores. In patients with depression, specific brain changes and evaluated IL-1β were identified, and the IL-1β interacted with right precentral gyrus to significantly affect the depressive symptoms. Our findings provide novel evidence indicating potential effects of inflammation on brain structure and behavior in NPC patients.

## Introduction

Nasopharyngeal carcinoma (NPC) is a malignant cancer which originates from the nasopharynx epithelium. NPC has a unique pattern of geographical distribution, with highest incidence rate in Southeast Asia, especially in Southern China (1). The routine treatment for NPC is the radiotherapy, with or without concurrent chemotherapy (2). Recent research reported that early-stage NPC patients who received intensive modulated radiotherapy (IMRT) could achieve excellent 5-year survival rate>90% (3). To improve the well-being and life quality of NPC survivors in the long-term recovery in cancer caring, growing attention is focused on the psychological status of patients, which is a key factor that may affect life quality.

Being depressive is one of the most common comorbid psychiatric symptoms in cancer patients, among which the incidence is approximately three to five times greater than that in the general population (4, 5). Depressed cancer patients have decreased compliance to cancer therapy recommendations (6), diminished quality of life (7) and increased morbidity and possibly, mortality (8). Previous studies found that depressive symptoms were related to decreased survival rate among patients with renal carcinoma (9) and gastric cancer (10). Thus, it is essential to identify psychopathological alterations and elucidate the underlying mechanisms in patients with NPC, which may extend our knowledge in such aspect and help develop effective psychological interventions.

In recent years, studies have indicated that inflammation is one of the shared bio-behavioral mechanisms in the pathophysiology of depression and cancer (11, 12). In contrast to the traditional notion that brain is an immunologically privileged site shielded behind the blood brain barrier, intensive work in the past 2 decades has indicated a complex interaction among the immune system, systemic inflammation and brain, which could result in alterations in mood and cognition (13). The tumor microenvironment contains various inflammatory immune cells such as macrophages, T and B lymphocytes, myeloid-derived suppressor cells, dendritic cells, natural killer cells, and neutrophils (14) that may promote tumor progression. Hence, peripheral-to-central inflammation may play a significant role in tumor-induced changes in behavior comorbidities such as depression. Accumulating evidence has shown that NPC patients have abnormal cytokine levels, including elevated levels of interleukin (IL)-6, IL-1 and tumor necrosis factor-alpha (TNF-α) as well as other molecules that are associated with inflammation process including soluble IL-2 receptor (sIL-2R), C-reactive protein (CRP) (15–17), and transforming growth factor-beta (TGF-β) (18, 19). It has also been demonstrated that elevated levels of peripheral pro-inflammatory cytokines or molecules, including IL-6, IL-1, TNF-α and CRP, are associated with the severity of depressive symptoms (20, 21). Meanwhile, clinical trials indicated that cancer patients administrated of the interferon alpha (IFN-α) or IL-2 were associated with the development of depressive symptoms during the therapeutic process (22). Furthermore, the anti-inflammatory interventions were effective in alleviating depressive symptoms and depression (23). The abovementioned evidence supports the hypothesis that the activation of the immune system may contribute to the development of depressive symptoms in NPC patients. However, how the depressive manifestations relating to the peripheral cytokine levels in patients with cancers and the potential underlying brain substrates remain largely unknown.

The progress of neuroimaging techniques provides opportunities for investigating the hypothesis of inflammation in the pathogenesis of depressive symptoms in NPC. Previous structural magnetic resonance imaging (MRI) studies explored the relationship between serum cytokine levels and brain morphology in healthy individuals or those with depression, and found that the hippocampal volume was inversely associated with the serum levels of IL-6, IL-1β and TNF-α (24, 25). These studies indicated that inflammation played a crucial role in neurotoxicity. However, the majority of prior studies focused on either specific regions of interest (ROIs) or a typical cytokine, and no studies hitherto have investigated cerebral structures and their associations with peripheral cytokine levels in NPC patients with depressive symptoms. Among the neuroimaging analyses, surface-based morphology (SBM) is suggested as a biologically informative approach with particular sensitivity to morphological changes of human brain (26). It allows the calculations of whole brain cortical parameters, including cortical thickness, surface area and volume with high robustness and repeatability (27).

Since pre-treatment research may inform biological consequences of tumor itself on the pathogenesis of cancer-associated psychological comorbidities without confounding effects of treatment (28), we included a group of untreated patients with newly diagnosed NPC in the present study. The purpose is to apply SBM approach to examine whether untreated NPC patients with depressive symptoms were accompanied by both abnormal cerebral morphology and changes of peripheral cytokines, and their potential relationships. We hypothesized that: (1) altered levels of serum pro-/anti-inflammatory cytokines would be detected in patients with NPC; and (2) patients would demonstrate cerebral structural abnormalities, that might be associated with altered serum cytokine levels and depressive symptoms.

## Materials and Methods

### Participants

The study was approved by the local ethics committee, and written informed consent was obtained from all subjects before participation. Twenty-nine newly diagnosed NPC patients without any treatment (mean age 43.41 ± 7.95 years) were recruited. All diagnoses were confirmed by histopathology later and all NPC patients were within stage T3N2M0 (stage T1:8, stage T2: 12, and stage T3: 9; N0:1, N1:8, N2: 20) as indicated by the 8th Edition of the American Joint Committee on Cancer (AJCC) Staging System. None of them had intracranial invasion or metastasis. For all included patients, this was the first time that they were diagnosed with NPC and they had not received any anti-tumor treatment before.

Forty-six healthy comparison individuals (mean age 41.20 ± 5.70 years) were recruited from same communities where the patients resided through poster advertisements. The patients and healthy comparisons were matched in age, sex, and years of education. All participants completed the Hamilton Rating Scale for Depression (HAMD). The demographics of patients and healthy comparisons are listed in **Table 1**.

**Table 1:** Demographic and clinical characteristics of the patients with nasopharyngeal carcinoma and healthy comparisons.

	NPC patients (N=29)		Healthy comparisons (N=46)		t/χ2	p
	Mean	SD	Mean	SD		
Age (years)	43.41	7.95	41.20	5.70	1.41	0.164
Sex (Male/female)	21/8		28/18		1.82	0.306
Education (years)	10.14	5.28	12.17	3.63	1.76	0.084
BMI	23.50	2.04	23.19	2.86	0.48	0.633
HAMD	5.50	3.63	1.97	0.51	5.76	<0.001
Serum cytokines						
IL-1β	29.98	13.56	20.77	12.38	2.99	0.004*
IL-2	17.09	6.54	10.98	3.74	5.12	<0.001*
IL-6	5.51	4.97	4.40	1.51	1.41	0.162
IL-8	5.55	2.67	4.77	0.69	1.89	0.063
IL-10	9.95	2.33	8.43	2.60	2.53	0.014*
IL-12	5.03	2.60	6.11	2.31	1.87	0.066
IFN-γ	52.70	20.65	46.11	12.68	1.7	0.093
TGF-β	1738.63	1232.82	1555.03	1244.59	0.62	0.539

BMI, Body Mass Index; NPC, nasopharyngeal carcinoma; HAMD, Hamilton Rating Scale for Depression; IFN, γ, interferon, gamma; IL, interleukin; TGF, β, transforming growth factor, beta; SD, standard deviation.

*indicated p values that survived from FDR correction.

Inclusion criteria for NPC patients were: (1) aging between 18 and 60 years; (2) with no intracranial invasion, no distant metastases, no prior substantial head trauma, no diabetes, no viral hepatitis, no positive human immunodeficiency virus status, or no other major medical illness; (3) receiving no chemotherapy, surgery or radiotherapy before; and (4) having no anti-inflammation drugs or therapeutics within a month prior to participation. Inclusion criteria for controls included no history of major psychiatric illness (as confirmed by the non-patient edition of the Structured Interview for DSM-V Axis I Disorders (SCID)), and without a first-degree relative with known history of major psychiatric or neurological illness. Exclusion criteria for all participants included: (1) previous radiotherapy of the brain; (2) existence of neurological disorders or other psychiatric disorders; (3) alcohol or drug abuse; (4) pregnancy; and (5) contraindications for MRI scanning.

### Data Acquisition

#### Blood Samples

The blood samples were collected before MRI scanning, but on the same day. Following overnight fasting for 8-12 hours, the participants were woken up at 8:00 am to collect the blood sample. All human venous blood samples, about 10 mL, were collected into two blood collection tubes using heparin as an anticoagulant, then centrifuged for 20 minutes at 1500r within 30 minutes of collection. The collected plasma samples were then stored at -80°C until the day of assay. All the blood sample were centrifuged before storing in the cold.

#### Brain MRI Data

The MRI examinations were performed on a 3 Tesla system (SIEMENS, Skyra, Germany) with a 20-channel phase array head coil. High-resolution T1-weighted images were acquired with a magnetization prepared rapid gradient echo (MPRAG) sequence (repetition time = 8.5 ms, echo time = 2.97 ms, flip angle = 9°, filed of view = 256×256 mm², matrix = 256×256, number of sagittal slices = 176, slice thickness = 1mm, voxel size = 1×1×1mm³, no slice gap). MR images were inspected by 2 experienced neuroradiologists to exclude subjects with gross abnormalities and visual scan artifacts in the images.

### Measurements of Cytokine Levels in Serum

A high sensitivity quantitative enzyme immunoassay technique (Q-Plex™ Custom Assay) was used to quantitatively measure serum levels of cytokine sin duplicate, including interferon-gamma (IFN-γ), IL-1β, IL-2, IL-6, IL-8, IL-10, and transforming growth factor-beta (TGF-β). The lowest limit of detection (LLOD) for the cytokines was 0.2 mg/L. Briefly, assays were calibrated and cytokine’s concentration was confirmed using the duplicate 6-points standard antigen curves. The blood sampling and the MRI scanning were performed on the same day. The average inter- and intra-assay coefficient of variation was <10%.

### Imaging Processing

The FreeSurfer package (version 6.0, http://surfer.nmr.mgh.harvard.edu/) was used to generate cortical reconstruction and volumetric segmentation of structural MRI data. This method has been shown with high test-retest reliability based on postmortem histological analysis and manual measurements. The reconstruction of the cortical surface mainly included skull-stripping, automated registration to Talairach space, subcortical gray/white matter segmentation, intensity normalization, tessellation of gray matter and white matter boundaries, automated topology correction and surface deformation to optimally place the gray/white and gray/cerebrospinal fluid borders defined at the location with the greatest shift in signal intensity (29–31). Individual surface maps were registered to a common average surface and analyzed after the interpolation steps. Vertex-wise cortical thickness was quantified as the nearest distance from the gray/white boundary to the pial surface, and cortical surface area was calculated as the mean area of the associated triangular region at each vertex. Cortical volume was defined as the product of cortical thickness and surface area. Additionally, subcortical segmentation and volumetric measurement of subcortical gray matter structures were performed, including thalamus, caudate nucleus, putamen, pallidum, hippocampus, amygdala and accumbens. Postprocessing visual inspection for quality of both imaging processes was conducted without knowledge of subject characteristics.

### Statistical Analysis

#### Demographics and Serum Cytokines

Two-sample t test was performed to compare age, years of education, body mass index (BMI) and HAMD scores between the NPC patients and healthy individuals, whilst Chi-squared test was used for sex distribution comparison.

With regards to different serum cytokines levels, t test was used to compare them between patients and controls, and false discovery rate (FDR) correction was adopted to preserve a p<0.05 experiment-wise threshold.

#### Intergroup Comparisons of Structural Brain Measures

The comparisons of brain anatomical measures between patients and healthy controls were conducted within the graphical interface of FreeSurfer-QDEC statistical tool. First, a smoothing step with 10 mm full width at half maximum (FWHM) Gaussian kernel was initiated to average the cortical thickness data across participants in the common spherical coordinate system, so that the inter-group structural differences can be measured using individual calculations. Then, a general linear model was performed for each vertex across the whole-brain to compare the cortical thickness, surface area and gray matter volume, adjusting for age, sex, and total intracranial volume. To correct for multiple comparisons, nonparametric cluster-wise correction was performed using Monte-Carlo simulation, and statistical significance was determined at a corrected cluster-level threshold of 0.05.

Given that the subcortical regions were segmented separately due to their special locations and only gray matter volume could be measured for them, the between-group differences in subcortical gray matter volumes were identified using the analysis of covariance (ANCOVA) implemented in the IBM SPSS 24 software (Armonk, NY, USA), with diagnosis as fixed factor and sex, age and total intracranial volume being the covariates. The level of statistical significance was set at p<0.05 after FDR correction across all examined subcortical structures.

#### Correlation analyses Between Altered Brain Measures, Cytokines, and Depressive Symptoms

We then further extracted average regional values of cortical thickness, surface area and gray matter volume with significant inter-group differences for each subject, and correlated them to the levels of serum cytokines that also differed between two groups. Additionally, the associations between brain structural abnormalities and the HAMD scores, and between levels of serum cytokines and HAMD scores were also examined in NPC patients. Statistical threshold for these correlation analyses was set at p<0.05 (2-tailed) after FDR correction.

Since we hypothesized that the tumor might cause informatory changes at the beginning, and then subtle but measurable alterations in brain happened thus caused depressive symptoms, we thus conducted a mediation analysis using altered serum cytokine as an independent variable, altered structural brain measure as the mediation variable and depressive symptom as the dependent variable. Mediation were determined using methods raised before (32) through three items: (1) the relationship between cytokine (independent variable) and depressive symptoms (dependent variable), (2) effects of cytokine (independent variable) on structural brain changes (mediator), and (3) effects of structural brain alteration (mediator) on depressive symptoms (dependent variable). If findings of all the three items are significant (p<0.05), we can conclude that structural brain alteration medicates the relationship between cytokines and depressive symptoms.

#### Subgroup Analysis

Since some NPC patients might not endorse depressive symptoms, the findings thus could be blurred by the heterogeneous samples. Therefore, we further conducted a subgroup analysis by dividing patients into those with depressive symptoms and those without in the exploratory analyses. This subgrouping was defined by setting the HAMD cutoff score at 7–a score no more than 7 indicating no depression, otherwise with depression (33). Finally, 8 patients with NPC were in depression subgroup while the other 21 were in the other subgroup, and the demographics and serum cytokines were presented in **Table S1**.

To determine whether depressive patients showed more severe brain changes than those who were not depressive, the altered brain measures observed in all patients were extracted and then compared between patient subgroups and healthy controls with ANCOVA and sex, age and total intracranial volume were controlled during the analysis. Mediation analysis was also conducted in the same way as noted above in depressive patient subgroup to examine the interaction between altered serum cytokines, structural brain changes and depressive symptoms.

## Results

### Demographics

There were no significant differences between NPC patients and healthy comparisons in age, sex ratio, years of education, or BMI (all p<0.05).

### Differences in Cytokines Levels Between NPC Patients and Healthy Controls

The patients with NPC showed significantly higher levels of IL-1β, IL-2 and IL-10 relative to healthy comparisons (all p<0.05 after FDR correction, **Table 1**), while there were no significant differences in serum levels of IFN-γ, IL-6, IL-8, IL-12 or TGF-β between the two participant groups (**Table 1**).

### Differences in Structural Brain Measures Between NPC Patients and Healthy Controls

**Figure 1:**
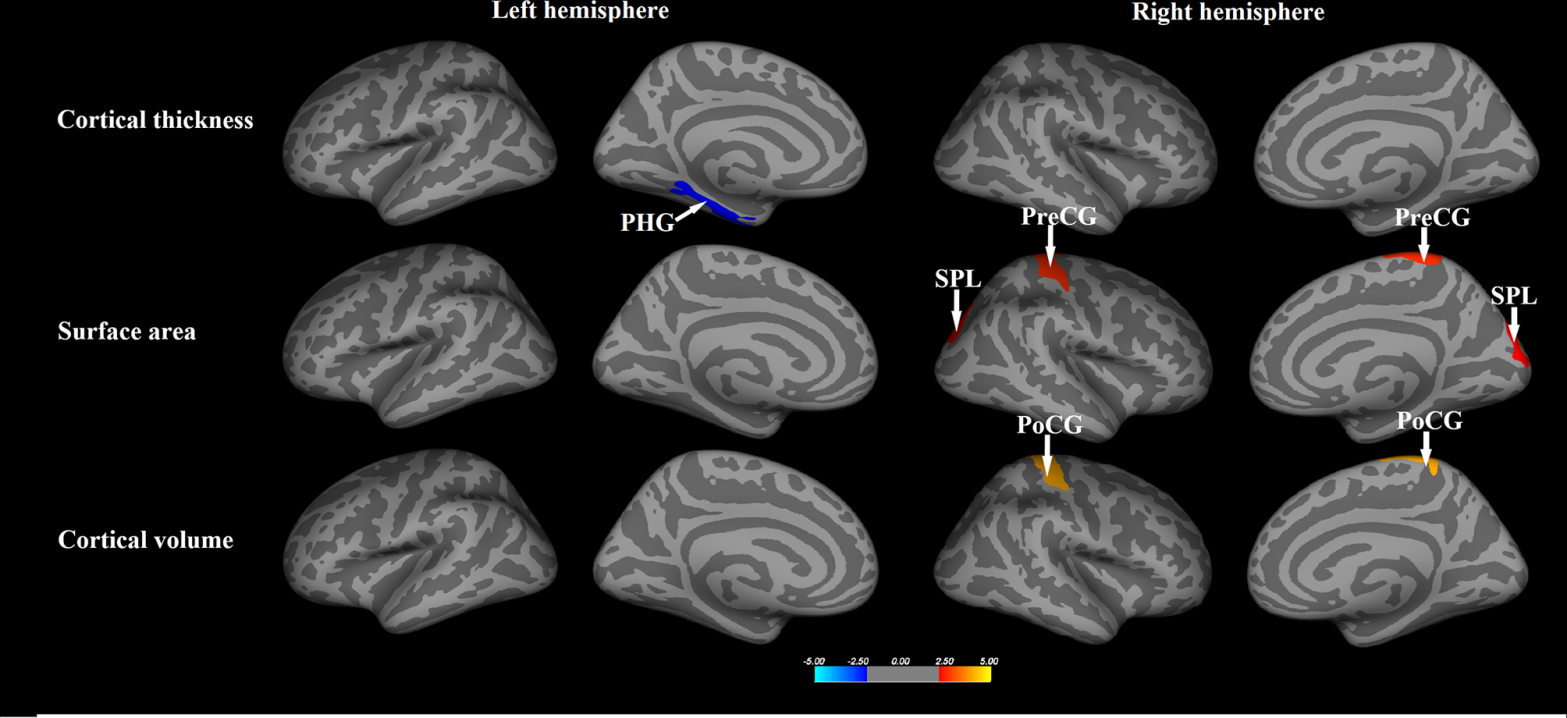
The whole brain comparisons of the cortical thickness, surface area and volume between patients with nasopharyngeal carcinoma and healthy comparisons. Orange or red clusters represent the NPC > HCs; blue clusters represent the NPC < HCs. PHG, parahippocampal gyrus; PreCG, precentral gyrus; PoCG, postcentral gyrus; SPL, superior parietal lobule.

In the SBM analysis, relative to healthy controls, the NPC patients showed significantly decreased cortical thickness in the left parahippocampal gyrus, but greater surface area in the right precentral gyrus and superior parietal lobule, and larger volume in the right postcentral gyrus (p<0.05, Monte-Carlo simulation, **Figure 1** and **Table 2**).

**Table 2:** Regional gray matter differences in cortical thickness, surface area, gray matter volume between patients with nasopharyngeal carcinoma and healthy comparisons.

Brain Regions	Measure	^a^Max	Talairach coordinates			Cluster size (mm^2^)	p^b^ for CWP
			x	y	z		
**Cortical regions**							
Parahippocampal gyrus. L	Thickness	-4.11	-23	-19	-24	1052.18	0.009
Precentral gyrus. R	Area	4.08	5	-28	66	1784.91	0.003
Superior parietal gyrus. R	Area	3.37	15	-83	30	1519.99	0.009
Postcentral gyrus. R	volume	4.03	5	-32	66	1865.21	<0.001
**Subcortical regions**		**NPC patients**		**Healthy comparisons**			
		Mean ± SD		Mean ± SD		**F**	**p**^c^
Thalamus. R	Volume	7457.23 ± 830.09		7095.67 ± 706.36		7.25	0.042
Caudate nucleus. R	Volume	3627.32 ± 353.71		3348.78 ± 451.47		12.61	0.014
Caudate nucleus. L	Volume	3618.78 ± 382.62		3355.09 ± 438.02		10.56	0.014

a Max: positive value means NPC patients> healthy comparison individuals; negative value means NPC patients< healthy comparison individuals.

b All regions survived cluster-wise correction for multiple comparisons (p<0.05).

c All regions survived false discovery rate (FDR) correction for multiple comparisons (p<0.05).

CWP, cluster-wise probability; SD, standard deviation; L, left; R, right.

With regards to subcortical gray matter volume, compared to control subjects, NPC patients also showed significantly larger volumes in the bilateral caudate nucleus and right thalamus (p<0.05, FDR corrected, **Table 2**).

### Correlations Between Altered Brain Anatomy, Serum Cytokines, and Symptomatology

Within regions with significant inter-group differences, significant negative correlation was found between level of serum IL-2 and the surface area of right superior parietal lobule (r=-0.53, p=0.003), and between level of serum IL-10 and the surface area of right superior parietal lobule (r=-0.40, p=0.031, **Figure S1**). The NPC patients also demonstrated significant positive correlation between level of serum IL-1β and HAMD scores (r=0.42, p=0.025, **Figure S2**), while the surface area of right precentral gyrus was negatively correlated with HAMD scores in the NPC patients (r=-0.427, p=0.024). Findings above were all corrected with FDR.

In the mediation analysis among measures noted above, no significant mediation effect was found of any brain anatomical change on the association between altered cytokine levels and depressive symptoms in NPC patients.

### Subgroup Analysis

The NPC patients with depression showed significantly higher levels of IL-1β and IL-2 relative to healthy comparisons, while patients without depression only had higher IL-2 than healthy subjects (p<0.05 after FDR correction, **Table S1**).

**Figure 2:**
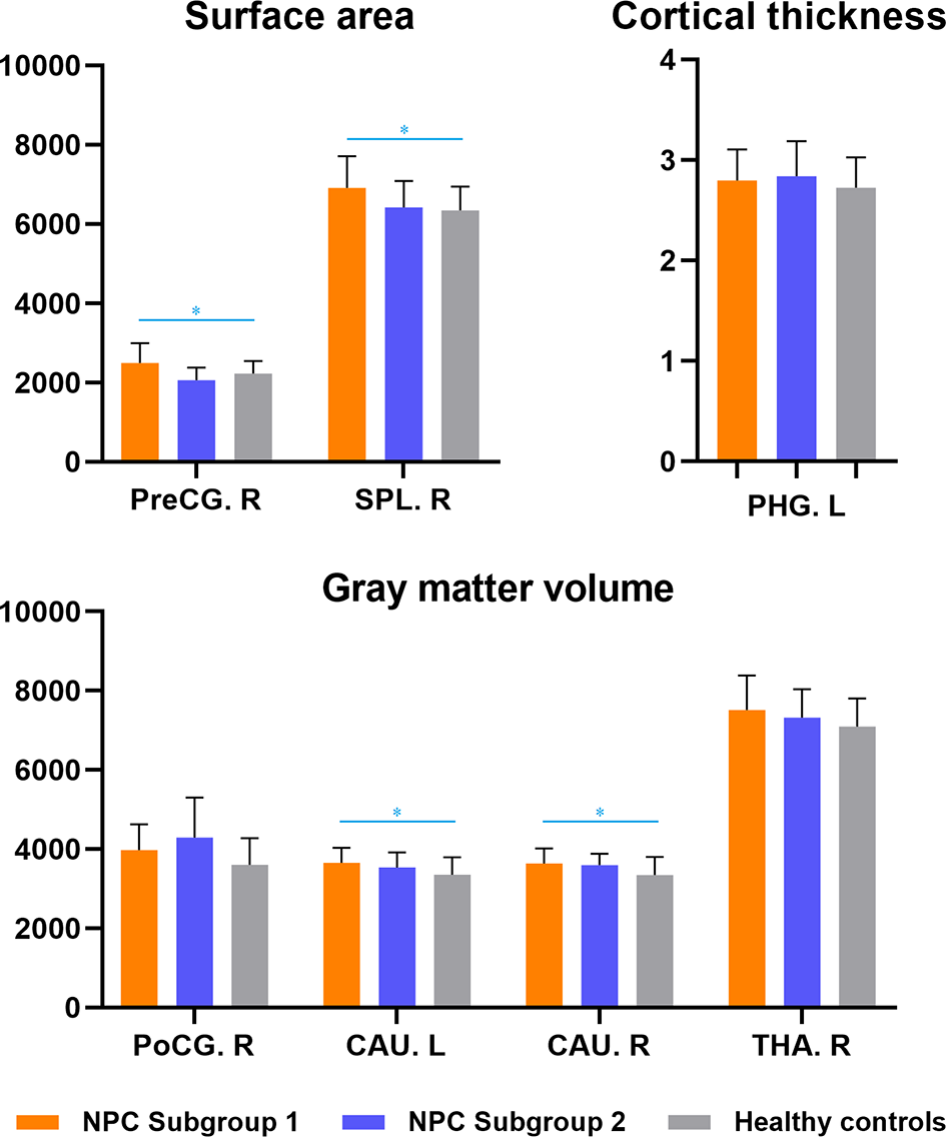
Subgroup analyses of structural brain abnormalities in nasopharyngeal carcinoma patients with and without depression. NPC Subgroup 1-patients with depressive symptoms; NPC Subgroup 2-patients without depressive symptoms. * indicated inter-group differences with statistical significance. PHG, parahippocampal gyrus; PreCG, precentral gyrus; PoCG, postcentral gyrus; SPL, superior parietal lobule; CAU, caudate; THA, thalamus; R, right; L, left.

The NPC patients with depression also exhibited significant larger cortical surface area in the right precentral gyrus and superior parietal lobule, as well as greater gray matter volumes in bilateral caudate, in contrast to healthy subjects, whereas no significant differences were observed between NPC patients without depression and healthy controls (**Figure 2**, **Table S2**).

In correlation analyses between depressive symptoms and cytokine levels or anatomical brain changes, a positive correlation between depressive symptoms and IL-1β (r=0.83, p=0.022) and a negative association between depressive symptoms and surface area of right precentral gyrus (r=-0.76, p=0.029) were observed. No significant mediation effect was found of this brain anatomical change on the association between IL-1β level and depressive symptoms, however, a multivariate regression analysis indicated that the levels of IL-1β and surface area of right precentral gyrus could exert significant effects simultaneously in predicting the depressive symptoms (beta=0.55 and -0.57, p=0.024 and 0.021, respectively).

## Discussion

To the best of our knowledge, this is the first study exploring the associations between altered serum cytokines, cerebral changes, and depressive symptoms in the NPC patients who had not been treated. Our findings revealed that patients not only had elevated levels of pro-inflammatory and anti-inflammatory cytokines after they were diagnosed with NPC, but also abnormal cortical and subcortical gray matter changes mainly involving limbic-striatal-thalamocortical circuits. More importantly, in subgroup analysis among patients with clinically-defined depression, the severity of depressive symptoms was significantly correlated to the levels of elevated cytokines and the subtle anatomical abnormalities simultaneously, and a multivariate regression analysis indicated that serum cytokines and brain structural changes might interact in some way to affect the depressive symptoms. These findings suggest that, in patients who were recently diagnosed with NPC and were emotionally affected, altered serum cytokine levels may play a pivotal role in interacting with the cerebral abnormalities and cause depressive behaviors.

Compared to healthy individuals, the NPC patients exhibited elevated levels of IL-1β, IL-2 and IL-10, which is in accordance with previous findings in patients with NPC (34, 35) and in tumor-bearing rodent models (36, 37). Moreover, in our subgroup analysis, patients with depressive symptoms had specific overexpression of IL-1β while patients without depression did not, and the evaluated IL-1β was also found with significant positive association with depressive symptoms. A prior study also showed that pro-inflammatory cytokines were elevated in cancer patients with comorbid depression relative to patients with cancer or depression alone (38). Our finding is also consistent with that elevated levels of IL-1β were associated with depressive-like behaviors such as anhedonia, psychomotor slowing, sleep disturbance, decreased social exploration, and reduced food/water intake in animal models (36, 37). Particularly, increased peripheral pro-inflammatory cytokines could enter central nervous system (CNS) *via* the leakage of blood brain barrier and lead to alterations in neurotransmission and neuroplasticity, contributing to the development of depression (39). These findings suggest an inflammatory process naturally happened in patients who affected with tumor, and the altered expression of IL-1β might be specific to the development of depressive symptoms.

In identifying subtle brain structural changes, relative to healthy subjects, all NPC patients and those with depressive symptoms exhibited both cortical and subcortical morphological abnormalities, primarily involving right precentral gyrus, right superior parietal lobule and bilateral caudate, while patients without depression did not show any significant change. Prior studies of cancer patients with depressive symptoms have identified significant lower brain activity and metabolic reductions in the frontal and parietal cortices, and basal ganglia, and reductions of these regions were related to the severity of depressive symptoms (40, 41). Structural studies also revealed that lower volumes in the caudate were significantly associated with higher degree of social anhedonia and depressive symptoms (42). Notably, the brain regions identified above were within the striatocortical pathway, which is important for the regulation of mood and associated cognitive, memory, motor and somatic behaviors (43) and therefore plays a critical role in the pathophysiology of depression. Our findings further enhanced the contribution of these morphological abnormalities of this circuit in causing depressive symptoms in NPC patients.

While the depressive symptoms were correlated with cytokines and brain changes, a multivariate regression analysis indicated that evaluated IL-1β and surface area of right precentral gyrus might interact in some way to significantly affect the depressive symptoms. As we noted in the results, the evaluated IL-1β was specifically found in depressive patients, these findings thus indicate that the inflammatory process might interact with the brain changes, and then contribute significantly to the development of depression. IL-1β is an important pro-inflammatory cytokine for inflammatory response, and physiological levels of IL-1β are previously found associated with the induction and maintenance of long-term potentiation, neurogenesis and neuroplasticity (44). Pathological IL-1β has been indicated to inhibit glutamate reuptake and lead to N-methyl-D-aspartate (NMDA)-mediated excitotoxicity by reducing expression of the presynaptic glutamate transporter, resulting in neuronal damage and structural abnormalities in depression (45). The region that might be implicated in the process above was the right precentral gyrus, and the structural and functional changes of this region have both been well noted in the in emotion regulation or cognitive-processing in depression (46, 47). The pathway of inflammation-brain-depression in patients with NPC is also consistent with the recently proposed mechanisms of neuroinflammation underlying the pathophysiology of psychiatric disorders including major depression (48). However, whether there is shared process of depressive symptom development between cancer patients and mental disorders requires more work in the future.

Several limitations of our study need to be acknowledged. First, the relatively small sample size may limit statistical power, especially when these patients were further divided into two subgroups with and without depressive symptoms. Therefore, the reported findings merit replications in future studies. Second, given the cross-sectional design and the correlational nature of employed analyses, none of the observed correlations in the present study can be interpreted as a reflection of causal relationship. Third, the examined peripheral levels of cytokines may not be sufficient to reflect the immune-inflammatory state in the CNS. Although we controlled for some important factors prior to blood draw that might influence inflammatory cytokines, effects of potential confounding factors such as smoking and BMI cannot be completely excluded. Further research with larger samples and longitudinal design is needed to clarify how inflammatory cytokines and brain anatomy interact to cause depressive symptoms in patients with NPC.

In conclusion, the present study revealed the alterations of serum cytokines and multiple cerebral structural abnormalities involving the striato-cortical circuits specially in NPC patients with depression. The alterations of inflammatory cytokines might interact with anatomical brain changes, and then cause depressive symptoms in these patients. These findings provided novel evidence relevant to the possible effects of inflammation on brain structure and behavior in newly diagnosed NPC patients.

## Data Availability Statement

The raw data supporting the conclusions of this article will be available on request from the corresponding authors, without undue reservation.

## Ethics Statement

The studies involving human participants were reviewed and approved by the Research Ethics Committee of West China Hospital of Sichuan University. The patients/participants provided their written informed consent to participate in this study.

## Author Contributions

SuL and YJ are responsible for the conception and design of the research. YW, WZ, XW, XL, SiL, GZ, and JL conducted the experiments. YW, WZ and SiL scored and analyzed the clinical and neuroimaging data. YW, WZ and XW conducted statistical analyses. YW, WZ, XW, SiL, QG, JL and SuL interpreted the experimental results. YW and WZ prepared figures and tables and drafted the manuscript. All authors revised the manuscript and approved the submission of the manuscript.

## Funding

This work was supported by the National Natural Science Foundation (Grant Nos. 81671664, 8212018014 and 82101998), Sichuan Science and Technology Program (Nos. 2021JDTD0002 and 2020YJ0018), the Science and Technology Project of the Health Planning Committee of Sichuan (Grant No. 20PJ010), Post-Doctor Research Project, West China Hospital, Sichuan University (Grant No. 2020HXBH005), the Fundamental Research Funds for the Central Universities (Grant No. 2020SCU12053), Postdoctoral Interdisciplinary Research Project of Sichuan University (Grant No. 0040204153248) and 1.3.5 Project for Disciplines of Excellence, West China Hospital, Sichuan University (Project Nos. ZYYC08001 and ZYJC18020).

## Conflict of Interest

The authors declare that the research was conducted in the absence of any commercial or financial relationships that could be construed as a potential conflict of interest.

## Publisher’s Note

All claims expressed in this article are solely those of the authors and do not necessarily represent those of their affiliated organizations, or those of the publisher, the editors and the reviewers. Any product that may be evaluated in this article, or claim that may be made by its manufacturer, is not guaranteed or endorsed by the publisher.
