# Supplementary material for: Cerebral Structural Abnormalities and Their Associations With Peripheral Cytokine Levels in a Group of Untreated Patients With Nasopharyngeal Carcinoma
Source: Front Oncol. 2021 Nov 26;11:740033. doi: 10.3389/fonc.2021.740033 (PMC8660672; doi:10.3389/fonc.2021.740033)
Supplement: Supplementary file 1 [file DataSheet_1.docx]

Supplementary Material

Cerebral Structural Abnormalities and Their Associations with Peripheral Cytokine Levels in a Group of Untreated Patients with Nasopharyngeal Carcinoma

**Supplementary Table 1**. Subgroup analysis of demographic/clinical variables and serum cytokines among nasopharyngeal carcinoma patients with and without depression and healthy comparisons.

| **Demographic and clinical variables** | **NPC Subgroup 1^a^ (N=8)** | **NPC Subgroup 2^b^ (N=21)** | **HC**  **(N=46)** | **Statistics** | ***p*** | ***Post hoc analysis*** | | |
| --- | --- | --- | --- | --- | --- | --- | --- | --- |
|  | **Mean (SD)** | | |  |  | **Subgroup 1**  **vs**  **Subgroup 2** | **Subgroup 1 vs HC** | **Subgroup 2 vs HC** |
| Age (years) | 45.38 (7.23) | 42.67 (8.25) | 41.2 (5.70) | F=1.467 | 0.237 | 0.422 | 0.072 | 0.400 |
| Sex (Male/female) | 4/4 | 17/4 | 28/18 | χ^2^=3.50 | 0.174 | 0.096 | 0.564 | 0.104 |
| Education (years) | 9.88 (3.09) | 9.28 (5.92) | 12.70 (3.83) | F=4.27 | 0.018 | 0.739 | 0.056 | 0.034 |
| BMI | 23.45 (2.57) | 23.52 (1.85) | 23.19 (2.86) | F=0.12 | 0.891 | 0.940 | 0.812 | 0.605 |
| HAMD | 9.13 (4.49) | 2.62 (1.43) | 1.97 (0.51) | F=55.40 | **<0.001*** | 0.299 | 0.003 | 0.058 |
| **Serum cytokines** | | | | | | | | |
| IL-1β | 37.15 (15.92) | 28.24 (12.11) | 20.77 (12.38) | F=6.37 | **0.003*** | 0.135 | **0.003*** | 0.027 |
| IL-2 | 19.16 (4.59) | 16.74 (7.04) | 10.50 (3.35) | F=17.68 | **<0.001*** | 0.407 | **<0.001*** | **<0.001*** |
| IL-6 | 4.60 (1.07) | 5.83 (5.76) | 4.31 (1.45) | F=1.44 | 0.244 | 0.585 | 0.608 | 0.258 |
| IL-8 | 4.89 (0.70) | 5.81 (3.13) | 4.69 (0.62) | F=2.73 | 0.072 | 0.456 | 0.441 | 0.131 |
| IL-10 | 10.60 (2.58) | 9.80 (2.31) | 8.29 (2.63) | F=4.09 | **0.021*** | 0.450 | 0.035 | 0.031 |
| IL-12 | 3.75 (3.11) | 5.36 (2.37) | 6.44 (1.97) | F=5.14 | **0.008*** | 0.165 | 0.063 | 0.064 |
| IFN-γ | 60.14 (24.74) | 50.37 (19.62) | 44.67 (11.34) | F=3.31 | 0.043 | 0.299 | 0.152 | 0.238 |
| TGF-β | 1939.26 (1062.98) | 1670.43 (1335.35) | 1540.87 (1254.90) | F=0.33 | 0.722 | 0.635 | 0.430 | 0.708 |

Abbreviations: NPC-nasopharyngeal carcinoma; HC-Healthy comparisons, BMI-Body Mass Index, HAMD-Hamilton Rating Scale for Depression, IFN-γ-interferon-gamma, IL-interleukin, TGF-β-transforming growth factor-beta, SD-standard deviation.

^a^ Subgroup 1, NPC patients with depressive symptoms; ^b^ Subgroup 2, NPC patients without depressive symptoms.

***** indicated p values that survived from false discovery rate (FDR) correction.

**Supplementary Table 2**. Subgroup analysis of structural abnormalities among patients with and without depressive symptoms of nasopharyngeal carcinoma and healthy comparisons.

| **Brain regions** | **Measures** | **NPC Subgroup 1^a^ (N=8)** | **NPC Subgroup 2^b^ (N=21)** | **HC**  **(N=46)** | **Statistics** | ***p*** | ***Post hoc analysis*** | | |
| --- | --- | --- | --- | --- | --- | --- | --- | --- | --- |
|  |  | **Mean (SD)** | | |  |  | **Subgroup 1 vs**  **Subgroup 2** | **Subgroup 1 vs HC** | **Subgroup 2 vs HC** |
| Parahippocampal gyrus. L | Thickness | 2.80 (0.31) | 2.84 (0.35) | 2.73 (0.30) | F=0.70 | 0.500 | 0.753 | 0.375 | 0.339 |
| Precentral gyrus. R | Area | 2492.91 (500.88) | 2056.63 (317.39) | 2222.98 (320.70) | F=5.22 | **0.008*** | 0.031 | **0.010*** | 0.181 |
| Superior parietal gyrus. R | Area | 6906.19 (798.43) | 6415.50 (671.41) | 6347.57 (592.48) | F=5.20 | **0.008*** | 0.135 | **0.002*** | 0.770 |
| Postcentral gyrus. R | volume | 3977.48 (648.06) | 4296.00 (1011.05) | 3605.67 (676.71) | F=4.33 | **0.017*** | 0.321 | 0.038 | 0.017 |
| Caudate nucleus. L | Volume | 3650.44 (387.08) | 3535.69 (382.87) | 3355.09 (438.02) | F=3.74 | **0.029*** | 0.480 | **0.010*** | 0.279 |
| Caudate nucleus. R | Volume | 3639.44 (381.27) | 3595.50 (288.98) | 3348.78 (451.47) | F=3.96 | **0.023*** | 0.771 | **0.013*** | 0.143 |
| Thalamus. R | Volume | 7510.51 (878.79) | 7317.39 (720.31) | 7095.67 (706.36) | F=2.20 | 0.118 | 0.585 | 0.043 | 0.418 |

Abbreviations: NPC-nasopharyngeal carcinoma; HC-Healthy comparisons, SD-standard deviation, R-right, L-left.

^a^ Subgroup 1, NPC patients with depressive symptoms; ^b^ Subgroup 2, NPC patients without depressive symptoms.

* indicated p values that survived from false discovery rate (FDR) correction.


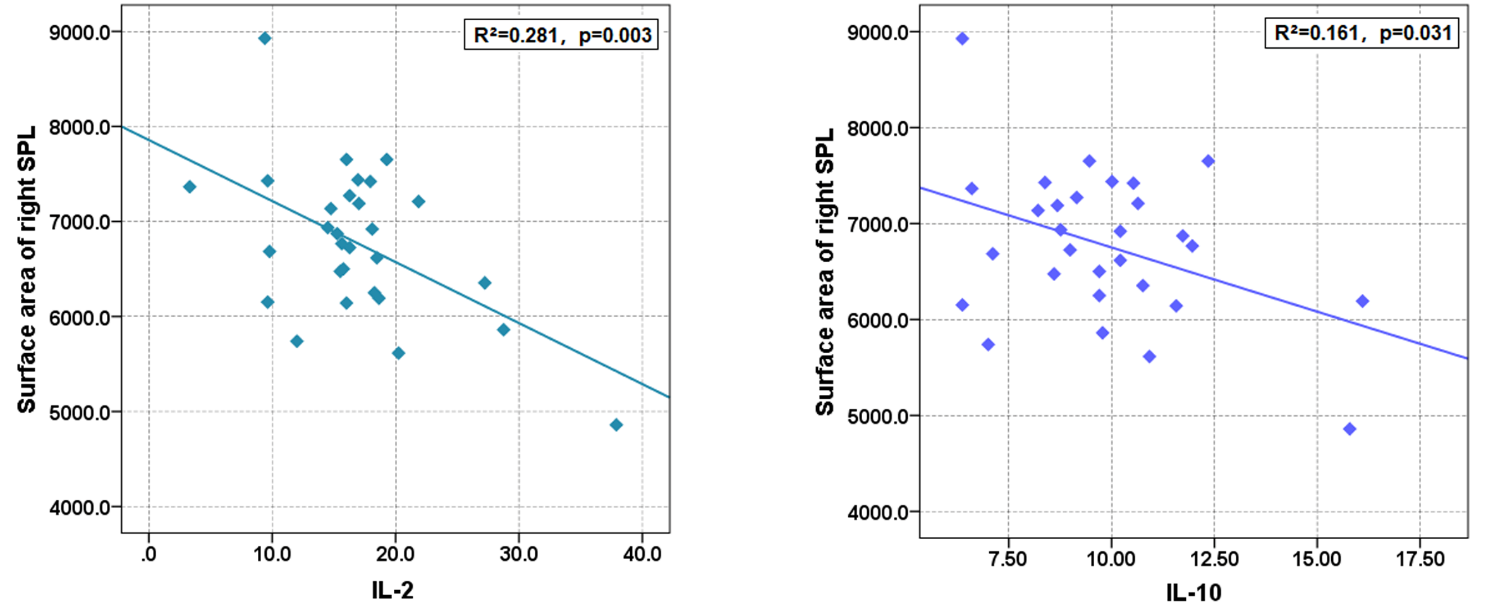


**Supplementary Figure 1.** Relationship between the surface area of right SPL and serum IL-2/IL-10 in patients with nasopharyngeal carcinoma. Abbreviations: NPC-nasopharyngeal carcinoma, SPL-superior parietal lobule.


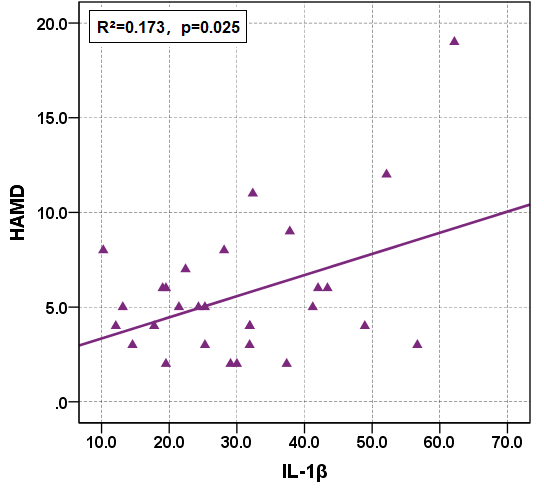


**Supplementary Figure 2.** Relationship between serum IL-1β and HAMD scores in patients with nasopharyngeal carcinoma. Abbreviations: NPC-nasopharyngeal carcinoma, HAMD-Hamilton Rating Scale for Depression.
